# St. Bartholomew's Hospital and Its Nurses

**Published:** 1912-06-22

**Authors:** 

**Affiliations:** Treasurer. St. Bartholomew's Hospital, London, E.C.


### THE EDITOR'S LETTER-BOX.

### St. Bartholomew's Hospital and its Nurses.

LORD SANDHURST'S STATEMENT.

*To the Editor of* THE HOSPITAL.

SIR,—In your last issue you mention the subject of the housing of the nursing staff at St. Bartholomew's Hospital, and you say that you know no reason why its improvement should be delayed. You add, and most truly, that I have the will to put the matter right. My willingness is shared by all the Governors, and our sole reason for delay is the want of funds.

Improvements in the nurses' accommodation have been and are being made from time to time, as far as the existing buildings will allow, but we realise that they are inadequate and that an entirely new home must be built as soon as possible.

The present appeal has not as yet yielded sufficient to pay off our debt to the bankers and to provide the additional income required to continue, without curtailment, the ordinary work of the hospital. For the present, therefore, we must live in hope of finding some beneficent person, or body of persons, willing to come to our assistance and so enable us to commence building a suitable home for our nurses.

If you would lend us the aid of your powerful influence towards this end, I should indeed be grateful.—I am, faithfully yours, SANDHURST, Treasurer.

St. Bartholomew's Hospital, London, E.C., June 17.

